# Mechanisms for synchronized burst firing in pyramidal cells using oscillatory inhibition: a model for attentional control

**DOI:** 10.1186/1471-2202-16-S1-P259

**Published:** 2015-12-18

**Authors:** Caroline Fischer, Paul HE Tiesinga, Marije ter Wal

**Affiliations:** 1 Department of Neuroinformatics, Donders Centre for Neuroscience, Radboud University Nijmegen, 6525 AJ, Nijmegen, the Netherlands

## 

Pyramidal cells have two different action potential initiation sites; one at the soma, which receives inputs from synapses contacting the basal dendrites or the soma, and one at the distal apical dendrite and tuft [1]. At the latter, calcium spikes can be elicited resulting in a plateau potential due to the influx of calcium that causes the pyramidal cell to fire bursts of action potentials [2]. This mechanism can be used to enhance the effect of top-down synaptic inputs that are received on the distal apical dendrite and tuft on activating its downstream targets. Calcium spikes can be elicited either by convergence of forward-propagating inputs at the tuft or by simultaneous depolarization from back-propagating action potentials and excitation at the tuft [3], referred to as FAC and BAC, respectively.

We investigated whether these two firing patterns can be synchronized by oscillatory inhibition in order to transmit top-down or combined top-down and bottom-up information to different brain areas. We hypothesize this mechanism as an explanation for the synchronization of burst spiking to distant local field potentials at beta frequencies (12-20 Hz) that was recorded in the prefrontal cortex and the anterior cingulate cortex during attentional control [4].

We simulated the behavior of pyramidal cells stimulated by oscillatory inhibition at the tuft and noisy excitatory inputs at the basal dendrite and tuft. First, we investigated the occurrence and phase-locking of FAC and BAC firing in one cell as function of the ratio of excitatory basal and tuft inputs. Second, we examined the synchronization of a population of pyramidal cells downstream to oscillatory inhibition with frequencies in the beta range. The pyramidal cells were modeled using four compartments and including calcium dynamics in the apical dendrite and tuft [5].

For single pyramidal cells FACs predominantly occur when the tuft receives stronger excitation than the basal dendrites, while BACs occur predominantly when the tuft and basal dendrites are excited equally strong. Both mechanisms have in common that they dominate the behavior of the pyramidal cell only when oscillatory inhibition is in the lower frequency range (2-20 Hz). For a 1:2 ratio between excitatory basal and tuft input FACs accounted for up to 85% of all spike events, whereas for a ratio of 1:1 BACs constituted up to 68%. Bursts activated by FACs and BACs were strongly phase-locked to the inhibitory drive, while bursts activated by other mechanisms had a lower coherence. The synchrony between pairs of pyramidal cells was quantified using the Schreiber measure [6] with the smoothing parameter set to σ=5 ms. When stimulated in the beta frequency range the synchrony between pairs of pyramidal cells was on average 0.78. Taken together, the results show that FAC and BAC firing can be used in conjunction with oscillatory inhibition to produce synchronized burst firing in pyramidal cells. This provides an effective means to transmit top-down or combined top-down and bottom-up information downstream.
